# Combination of Intracardiac Echocardiography and Contact Force Sensing for Left Ventricular Papillary Muscle Arrhythmias

**DOI:** 10.3390/jcm12093154

**Published:** 2023-04-27

**Authors:** Tongshuai Chen, Lujie Chang, Bing Rong, Kai Zhang, Guanqi Fan, Jing Kong, Mingying Ling, Qingyu Kong, Kellina Maduray, Cuifen Zhao, Jingquan Zhong

**Affiliations:** 1 National Key Laboratory for Innovation and Transformation of Luobing Theory, The Key Laboratory of Cardiovascular Remodeling and Function Research, Chinese Ministry of Education, Chinese National Health Commission and Chinese Academy of Medical Sciences, Department of Cardiology, Qilu Hospital of Shandong University, Jinan 250012, China; 2 Department of Cardiology, Qilu Hospital (Qingdao), Cheeloo College of Medicine, Shandong University, Jinan 250012, China; 3 Department of Pediatric Cardiology, Qilu Hospital of Shandong University, Jinan 250012, China; 4 Department of Geriatric Medicine, Qilu Hospital of Shandong University, Jinan 250012, China; 5 Department of Radiology, Qilu Hospital of Shandong University, Jinan 250012, China

**Keywords:** papillary muscles, left ventricular arrhythmias, catheter ablation, intracardiac echocardiography, contact force sensing

## Abstract

Objectives: The catheter ablation of ventricular arrhythmias (VAs) arising from the left ventricular (LV) papillary muscles (PMs) is challenging. This study sought to address whether the combination of intracardiac echocardiography (ICE) and contact force sensing (CFS) can improve the acute and long-term ablation outcomes of left ventricular papillary muscle arrhythmias. Methods and Results: From May 2015 to August 2022, a total of thirty-three patients underwent catheter ablation for LV PM arrhythmias: VAs were located in anterolateral PMs in 11 and posteromedial PMs in 22. A combination of intracardiac echocardiography (ICE) and contact force sensing (CFS) was used in 21 of the 33 procedures. A mean of 6.93 ± 4.91 for lesions was used per patient, comparable between the CFS/ICE and no ICE/CFS (4.90 ± 2.23 vs. 10.17 ± 5.89; *p* = 0.011). The mean CF achieved in the ICE/CFS group was 7.52 ± 3.31 g. Less X-ray time was used in the combination group (CFS/ICE: 165.67 ± 47.80 S vs. no ICE/CFS: 365.00 ± 183.73 S; *p* < 0.001). An acute success rate of 100% was achieved for the ICE/CFS group (n = 22) and 66.67% for the no ICE/CFS group (n = 8). VA recurrence at the 11.21 ± 7.21-month follow-up was 14.2% for the ICE/CFS group and 50% for the no ICE/CFS group (*p* = 0.04). No severe complications occurred in all patients. Conclusions: The combination of intracardiac echocardiography (ICE) and contact force sensing (CFS) could provide precise geometries of cardiac endocavitary structures and accurate contact information for the catheter during ablation, which improved acute and long-term ablation outcomes. The routine adoption of this strategy should be considered to improve the outcomes of LV PM VA ablation.

## 1. Introduction

The left ventricular (LV) papillary muscle (PM) is increasingly being recognized as a source of ventricular arrhythmias (VAs) in patients with or without structural heart diseases [1,2]. Premature ventricular contractions (PVCs) originating from the papillary muscle (PM) have recently been described as a distinct clinical entity with peculiar features [3]. In addition, PVCs arising from the PM may play a role as triggers of ventricular fibrillation (VF) [4,5].

Although catheter ablation is increasingly being used for treating LV PM VAs, it is still challenging for a variety of reasons; anatomically, the PM originates from the mid or apical one-third of the LV, and protrudes like fingers into the LV cavity [6]. Recently, the term ‘fourth dimension’ has been proposed to describe the endocavitary structures such as the moderator band, false tendons and PM. These structures are all potential arrhythmogenic foci and are difficult to target during ablation due to their complex 3D geometry [7]. Histologically, the PM is composed of ventricular myocytes and a rich subendocardial network of Purkinje fibers covered with a layer of endothelium, which may account for a single origin with preferential conduction to multiple exit sites [8]. Idiopathic VAs from PMs are frequently sensitive to catecholamines, noninducible through programmed stimulation and nonentrainable, which suggests triggered activity or abnormal automaticity as the electrophysiological mechanism [9]. Therefore, several radiofrequency (RF) lesions on different parts of the PM are required to completely eliminate the VAs. Functionally, excessive ablation may cause potential valve regurgitation and other complications [10,11]. For the above-mentioned issues, ablation outcomes were less favorable compared with other locations, such as the outflow tract [12]. Recently, new technological innovations have brought new insights regarding the complexity of PM arrhythmias, with Riccardo et al. reporting an echofacilitated 3D electroanatomic mapping technique that allows for the real-time creation of precise geometries of cardiac chambers, which would be useful for the catheter ablation of VAs originating from the PM [13]. Contact force sensing (CFS) technologies were also shown to improve PM VA ablation outcomes [14]. However, there is insufficient evidence that the combined use of intracardiac echocardiography with contact force sensing can improve PM VA ablation outcomes. In our study, the acute and long-time success rates of LV PM VA ablation were compared with different technique applications.

## 2. Methods Section

### 2.1. Patient Characteristics

**Figure 1:**
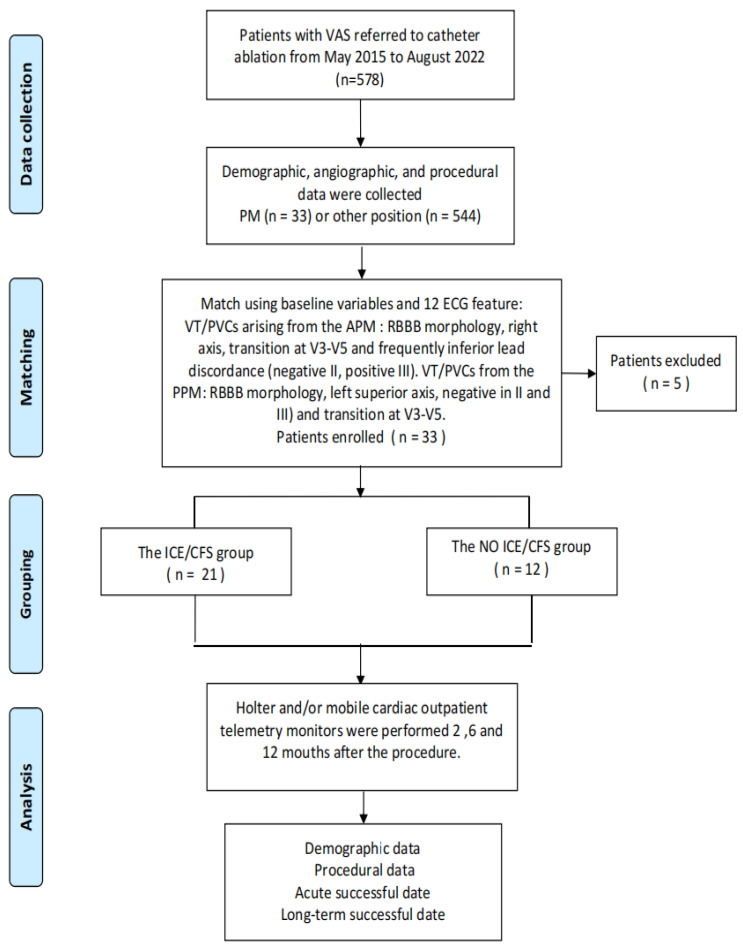
The basic process of the experiment was briefly described, divided into four main stages: data collection, matching, grouping and analysis.

Thirty-three patients with LV PM arrhythmias were identified from a retrospective review of 578 consecutive patients with symptomatically sustained ventricular tachycardia (VT), nonsustained VT or PVC in the Qilu Hospital of Shandong University from May 2015 to August 2022. All patients underwent electrophysiological studies and ablation, following institutional practice (trial number ChiCTR2100052256). Patients with right ventricular (RV) PM VAs were excluded from the study (Figure 1). The study was approved by the institutional review committee and all subjects gave written informed consent. A detailed medical history was collected at baseline and a careful analysis of the electrocardiogram was performed before the procedure (Table 1).

The patients were divided into two groups according to whether the combination of ICE and CFS was used during the ablation. The mapping of the no ICE/CFS group was performed using a 3.5 mm open-irrigation-tip catheter (Thermacool Navi Star; Biosense Webster Inc.). The ablation technique was selected according to the operators’ preferences and available technologies. The clinical data of the samples were obtained from the database of the Qilu Hospital and provided by the patients themselves. All transthoracic echocardiograms, electrocardiograms (ECG) and 24 h Holter laboratory test result recordings at baseline and throughout the follow-up were reviewed. The research samples and data included in this study were reviewed and approved by the ethical audit committee of the Qilu Hospital of Shandong University (no. KYLL-202111-041). All methods were carried out in accordance with relevant guidelines and regulations.

### 2.2. Electrophysiology Study, Mapping and Ablation

Antiarrhythmic medications were discontinued at least 5 half-lives before the procedure, and the electrophysiology study was conducted under minimal sedation. Intravenous heparin was used to maintain an activated clotting time greater than 300 s during the LV mapping and ablation.

#### 2.2.1. Intracardiac Echocardiography

**Figure 2:**
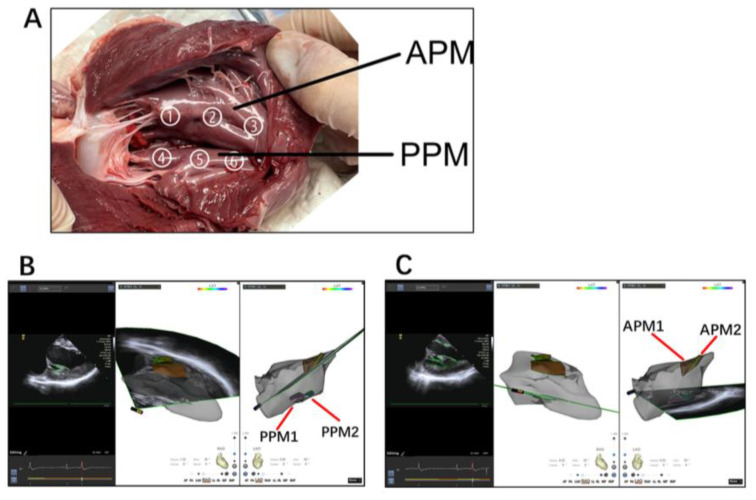
(**A**) An autopsy specimen of a pig heart exhibiting the distribution of the left ventricular papillary muscle; no. ① and ④ show the distal third of the PM (tip); no. ② and ⑤ show the middle portion of the PM (body); no. ③ and ⑥ show the proximal third of the PM (base). (**B**) APM and PPM were visualized in each 2D plane, including the LV endocardium; both heads of the papillary muscles were marked with different colors and delineated to create a detailed 3D model; two heads were separately described as APM1 (brown, the septal side) and APM2 (yellow, the freewall side), and two heads of the PPM, PPM1 (pink, the septal side) and PPM2 (green, the freewall side) (**B**,**C**).

**Figure 3:**
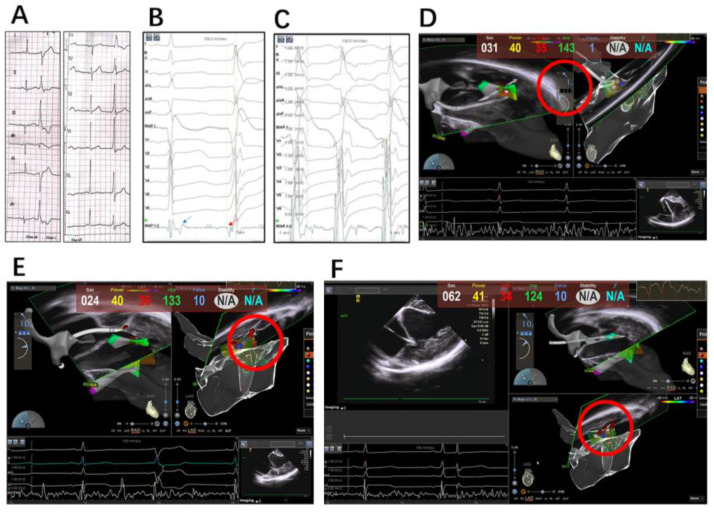
(**A**) Representative PVC body ECG of APM; (**B**) ECG of sinus rhythm and clinical PVC at ablation site; late potential seen during sinus rhythm (blue arrow) when reversed during PVC (red arrow) and preceded it by 32 ms; (**C**) pace mapping at the site of blue ball in (**D**) produced best pace; (**D**) catheter through retroaortic access was visualized on tip of anterior papillary muscle with ICE, but the CFS showed only 1 g; (**E**) catheter through trans-septal access gained better CFS, but failed to eliminate PVC completely where the vector was from above to below. (**F**) Clinical PVC was suppressed with contact orientation adjusted from below to above.

**Figure 4:**
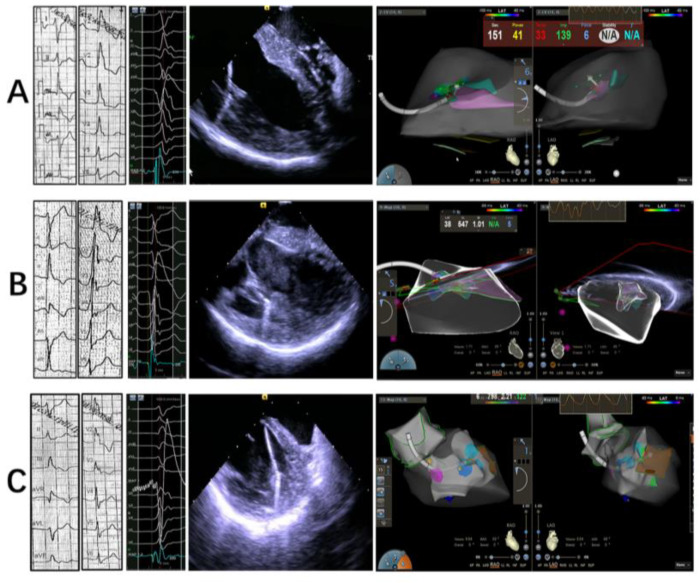
Representative ECG, intracadiacechography and 3D electroanatomical mapping at tip, body and base of APM, respectively (**A**–**C**).

**Figure 5:**
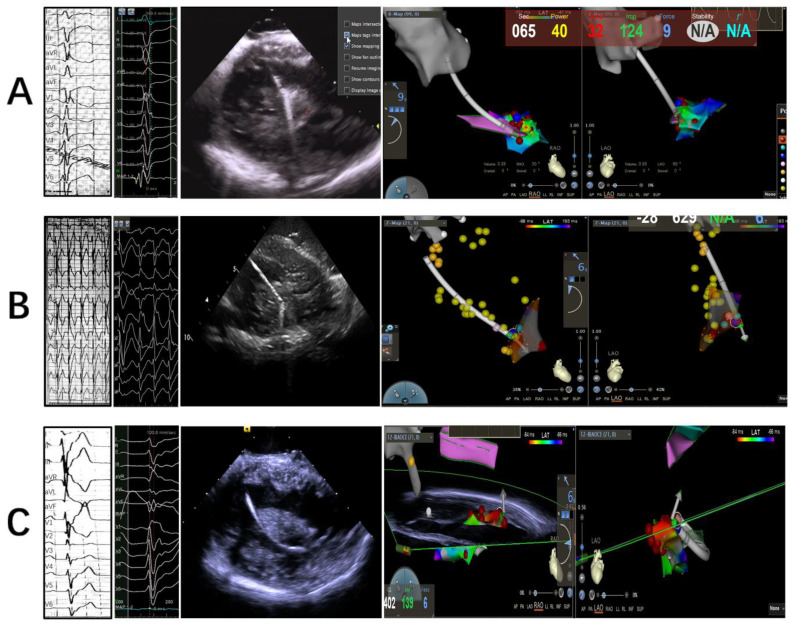
Representative ECG, intracadiacechography and 3D electroanatomical mapping at tip, body and base of PPM, respectively (**A**–**C**).

An ICE catheter (SOUNDSTAR^®^ Catheter, Biosense-Webster) was advanced through the femoral vein and positioned in the RV to model the different LV structures, as previously described [13]. By manipulating the ICE catheter, a 3D electroanatomic map of the LV was generated using the CARTO system (Biosense Webster Inc., Diamond Bar, CA, USA). The PM and other endocavitary structures were reconstructed from the ICE integration using the CARTOSound Module (Biosense Webster Inc.). Three segments were attributed to each PM: the tip, at the point of the insertion of the chords (distal third of the PM); the body (middle portion of the PM); and the proximal third of the PM as the base, which was in continuity with the LV inferior wall (Figure 2A). The models of the anterolateral papillary muscles (APMs) and posteromedial papillary muscles (PPMs) were further constructed on the transverse sector, with both APMs and PPMs typically having two heads (the septal one and the freewall one), each of them marked with different colors (Figure 2B,C). The localization of PM VAs relied mainly on the activation mapping of the clinical VT/PVCs (Figure 3); the main requisite for mapping was the presence of spontaneous or inducible VT/PVCs and the ability to image the PMs in real-time with ICE, which was essential to ensure adequate catheter–tissue contact and the correct orientation of the catheter tip during the mapping and ablation (Figure 4 and Figure 5).

#### 2.2.2. Mapping and Ablation

Both a retroaortic and trans-septal access to the LV could be used during the procedure. The retroaortic access was mostly approached, while the lateral aspect of the APM was approached in a trans-septal fashion. Detailed activation mapping was used in all cases to define the VA site of origin as follows: the catheter was manipulated under ICE and 3D electroanatomic map guidance to sample all heads of the PM to identify the earliest ventricular activation sites. Lesions of ablation included sites with ventricular activation earlier than −30 ms preQRS and a QS pattern in the local unipolar recording; the site of a successful ablation sometimes exhibited a late potential in the sinus rhythm that became presystolic during the PVC. Pace mapping was routinely performed in order to identify the site with the best pace map. Sites of best pace were usually defined as an excellent pace map (≥11/12 leads) (Figure 3A–C).

Radiofrequency ablation was delivered at the site of earliest activation and/or with the best pace map. Efforts were devoted to achieving at least 5 g of contact force at the site of energy delivery. Power was initiated at 30 W and then increased to a maximum of 45 W to achieve an impedance drop of 10 Ω with a temperature limit of 43 °C. When an acceleration or reduction in the incidence of PVC was observed, RF delivery was continued for 60 s, and a second application of RF was delivered; if there was no change in VA behavior after <30 s of RF delivery, then the lesion was terminated and the catheter was repositioned. During several ablations around the site of earliest activation, alternative contact directions were attempted with vector orientations of the catheter tip. Additionally, the trans-septal approach was adopted if the ablation contact force was not sufficient with the retroaortic access (Figure 3D–F).

### 2.3. Outcomes and Follow-Up

Acute success was defined as a targeted clinical VA elimination and noninduction for at least 30 min after ablation with isoproterenol infusion and ventricular pacing agitation. A postprocedural follow-up was based on symptom recurrence and Holter monitoring. Patients were evaluated 2 months after the procedure in the outpatient clinic, and contacted us in time if clinical symptoms reoccurred. Holter and/or mobile cardiac outpatient telemetry monitors were performed 6 and 12 months after the procedure. For patients who were not followed-up with at our facility, the referring cardiologist or the patients were contacted to obtain information on any devices (ECG/Holter), including clinic visits and telephone calls. A successful long-term catheter ablation was defined as a significant reduction (reduction in the clinical VA by ≥85%) or absence of clinical arrhythmia during the follow-up. In our study, no ICDs or implantable Holter devices were implanted after the ablation.

### 2.4. Statistical Analysis

Continuous data were expressed as mean ± SD, and categorical data were expressed as the number (%). Categorical variables were expressed as numbers and percentages. Continuous variables were compared using the unpaired Student’s *t*-test (parametric) or Mann–Whitney U-test (nonparametric). Kaplan–Meier curves were generated and a comparison between the two groups was performed using the log-rank test. The categorical variables were compared using the chi-square test or Fisher exact test. Statistical analyses were performed by using IBM SPSS version 24.0 (IBM SPSS Statistics, IBM Corporation, Armonk, NY, USA), and statistical significance was defined as a 2-sided *p* value of <0.05.

## 3. Results

### 3.1. Baseline Characteristics

A total of thirty-three patients were included in our study. Overall, the mean age was 49 ± 15 years and 69.7% were male. Twenty-five patients (75.7%) manifested isolated premature ventricular complexes (PVCs), eight (24.2%) had nonsustained VTs and the mean duration of symptoms was 19.91 ± 42.68 months. Eight patients (24.2%) had hypertension and thirteen patients (39.4%) had a history of smoking. Structural heart disease was present in 12 patients (36.4%) and the mean ejection fraction for the entire cohort was 58 ± 8%. The mean left ventricular diameter (LVD) overall was 51.00 ± 5.33 mm, and the mean left atrial diameter (LAD) was 38.38 ± 5.96 mm. No patients had an MV prolapse. In addition, we recorded the myocardial enzyme profile before the patients underwent the ablation, including the mean creatine kinase-MB (CK-MB) 2.29 ± 6.25 ng/dL, creatine kinase (CK) 110.45 ± 180.88 ng/dL, NT-proBNP 449.73 ± 753.40 ng/mL and cardiac troponin I (CTNI) 1.82 ± 1.66 ng/dL. Twenty-four patients took AADs preoperatively and twenty-five postoperatively. No significant differences in baseline characteristics were noted between both study groups (Table 1).

### 3.2. Procedural Characteristics

ICE/CFS ablation catheters were used in 21 (63.6%) cases, whereas non-CFS/ICE catheters were used in 12 (36.3%). Overall, VAs were targeted at the posteromedial PM (PPM) in 22 patients (66.7%) and at the anterolateral PM (APM) in 11 patients (33.3%), with VAs originating from the PM body in 11/21 (52.38%) patients in the ICE/CFS group. The retrograde aortic access approach was undertaken in 22 patients (66.7%) and combined access in 11 (33.3%) patients. At the site of a successful ablation, the earliest ventricular activation preQRS was achieved with an average of 35.10 ± 5.08 ms for the ICE/CFS group and 34.25 ± 4.35 ms for the no ICE/CFS group (*p* = 0.618). During the RF energy delivery, the mean CF achieved in the ICE/CFS group were 7.52 ± 3.31 g. The total amount and distribution of lesions are described in Table 2. A mean of 6.93 ± 4.91 lesions was used per patient, comparable between the CFS/ICE and no ICE/CFS (4.90 ± 2.23 vs. 10.17 ± 5.89; *p* = 0.002). The mean procedure time was 157.64 ± 55.75 min (CFS/ICE: 157.38 ± 49.38 min vs. no ICE/CFS: 158.08 ± 67.88 min; *p* = 0.973) and mean RF application time was 274.45 ± 138.96 S (CFS/ICE: 258.24 ± 141.39 vs. no ICE/CFS: 302.83 ± 135.80; *p* = 0.405). On average, 247.5 ± 160.75 s total X-ray time was used per ablation (CFS/ICE: 165.67 ± 47.80 S vs. no ICE/CFS: 365.00 ± 183.73 S; *p* < 0.001).

### 3.3. Outcome and Follow-Up

**Figure 6:**
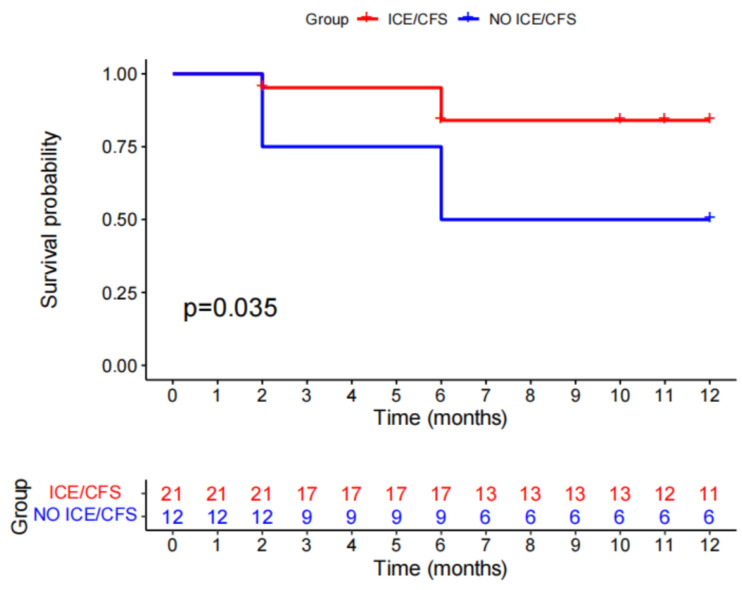
Kaplan–Meier survival estimates for recurrence.

The acute success rate was 29/33 (87.87%), with a tendency for a higher success rate in patients whose first procedure was performed with compared to without ICE/CFS (21/21 100% vs. 8/12 66.67%, *p* = 0.012) at a mean follow-up of 11.21 ± 7.21 months, whereas the clinical success rate was 24/33 (72.72%) in all cases (ICE/CFS:18/21 85.8%; no ICE/CFS: 6/12 50.00%; *p* = 0.044) (Table 2 and Table 3). Figure 6 shows patients free from ventricular arrhythmia after catheter ablation. The cost performance was apparently different between the two groups (no ICE/CFS: 4519.03 ± 1504.44 USD; ICE/CFS: 7864.86 ± 769.79 USD; *p* < 0.001).

## 4. Discussion

Catheter ablation is increasingly being used for treating LV PM VAs, but it can be challenging to perform for a variety of reasons, including due to anatomic complexities of the PM, variable locations of the VA sites and changing exits during ablation. A lot of effort has been contributed through technological innovations, as well as methodological improvements. Catheter ablation is an effective and safe strategy for the treatment of PM VAs, with an acute success rate of 88.1%, a long-term success rate of 69.2% and a relatively low procedural complication rate of 1.7% [15]. The salient findings of our study were as follows: (1) the combined use of intracardiac echocardiography and contact force sensing allowed for the precise mapping of each PM head; (2) contact force sensing was complementary in evaluating tissue contact besides ICE; (3) performing ablation lesions guided with vector orientation around the earliest site was useful for the elimination of PM arrythmias entirely.

### 4.1. Integration of Echocardiography and 3D Mapping

Ultrasound imaging can be integrated with electroanatomic mapping, and has been used in the catheter ablation of atrial fibrillation [16]. Echofacilitated 3D electroanatomic mapping seemed to be more indispensable when performing catheter ablation for arrhythmias from endocavitary structures, such as the PM, moderator band and false tendons. It was suggested that the integration of echocardiography may improve the feasibility of this type of procedure for several reasons: (i) both computed tomography and echocardiography integration into the mapping system were suitable for this type of structures; however, echocardiography was preferable, because it could provide a real-time direct visualization of the catheter’s position and papillary muscles; (ii) the provision of detailed anatomical structures, such as the separated heads of PMs, to improve the navigation of the ablation catheter; (iii) the monitoring of contact between the catheter tip and the PM during pace mapping, activation mapping and ablation. In our study, less fluoroscopy times and numbers of lesion were used in the ICE/CFS group, and we also found the ICE-generated 3D cardiac anatomy useful in understanding the lesion distributions relative to different segments of the targeted PMs. The PM body (11/21, 52.38%) was the most frequent site of effective lesions in the ICE/CFS group. In our study, no preprocedural cardiac MRIs were performed; however, 6/21 (28.50%) showed the presence of hyperechogenicity on ICE. A recent meta-analysis, including 19 studies with 2186 patients (with a history of either atrial or ventricular arrhythmias), similarly showed that the use of ICE was associated with a significantly reduced fluoroscopy time, fluoroscopy dose and procedural time, as compared to those performed without ICE [17]. The use of ICE required another femoral vein access to implant the 10F catheter, and no related complications were found in our study. In addition, a new type of 8F ICE catheter could further reduce the risk of venous complications. Goya et al. also showed that venous access complications appeared numerically higher for ICE over the comparator. However, the difference between the groups was not statistically significant (RR 1.93; *p* = 0.14) [17].

### 4.2. Contact Force

A further challenge for PM VA ablation is that consistent favorable contact is difficult to achieve for the duration of lesion application on highly mobile endocavitary structures such as the PM. Although ICE could monitor the contact between the catheter tip and the PM, contact force sensing, which has been proved to be effective in achieving durable chronic pulmonary vein isolation in atrial fibrillation ablation, is still in debate for use in PM arrhythmias. Aung N. Lin et al. reported that the use of CFS technology did not influence acute and long-term outcomes in an observational study, and inferred that the lack of benefits from CFS may be related to the consistent use of ICE guidance, which was used in all catheter ablations; however, it was noted that the average CF achieved during the catheter ablation of LV PM VAs was ~11 g; therefore, it was reasonable to speculate that the satisfactory ablation outcomes also benefited from contact force sensing [18]. In our study, the average contact force was 7.52 ± 3.31 g in the ICE/CFS group; furthermore, in some cases in our study, we observed an acute success ablation for PM PVCs within 5 s when the contact force indicated 1.0 g (Figure 4C, Video S1), though ICE showed contact between the catheter tip and APM; however, the PVC recovered 2 weeks after the ablation procedure. There were discrepancies between the two methodologies (the CFS value and intracardiac echography image), and although the catheter tip-to-tissue contact could be observed with ICE clearly, the CFS value was only 1 g. Therefore, the combination of CFS and ICE was complementary in the ablation of PM VAs. Tobia et al. also stressed that CF-sensing catheters provided continuous, real-time information about the catheter–tissue CF, which is a key determinant of lesion size [19].

### 4.3. Optimizing Ablation Lesions

Often, several RF lesions on different parts of the PM are required to completely eliminate the VT/PVC, with ablation outcomes likely enhanced through the use of more sophisticated methods for optimizing lesion efficacy, incorporating metrics beyond CF. A previous study by Wo et al. reported that if the first one or two shots of RFCA at the initial targeted site failed, they would adopt a circumferential approach with multiple ablations applied to the base of the PMs to eliminate all VAs completely [20]. In an ex vivo viable bovine model, Udi Nussinovitch et al. found that a catheter orientation perpendicular to the PM tissue achieved more effective and larger ablation lesions, with greater lesion depth. This may have implications for the chosen ventricular access approach, the type of catheter used, consideration for remote navigation and steerable sheaths [21]. Our study shared the same opinion; around the site of earliest activation, alternative contact directions were attempted, guided by the vector orientation of the catheter tip; additionally, the trans-septal approach was be considered if the retroaortic access was firstly approached (Figure 3D–F; Videos S2–S4). It was also suggested that high power is useful, as it may overcome the challenges imposed by poor stability [22]. In our study, the catheter orientation and force directionality for the PM VA ablation presented advantages; if the first shot at the target site failed or the contact force was insufficient, alternate contact was guided with vector orientation around the earliest site. Furthermore, trans-septal access was considered if the retroaortic access was approached first. The adoption of this strategy could achieve acute and long-term success with less X-ray time and lesions.

### 4.4. Limitations

As this was a single-center retrospective study of the catheter ablation of PM-related arrhythmias, the catheter ablation strategy used here may not be generalizable to all patients with LV PM arrhythmias. A prospective validation cohort study, ideally with multicenter participation, could validate our findings. Furthermore, the small number of patients in our report did not allow for an exhaustive statistical assessment. This was partly due to the fact that patients with PM arrhythmias represent a small subset of subjects referred for ablation, making them less suitable for larger studies. This was not a randomized trial; thus, the conclusions were only hypothesis-generating, and related to the feasibility rather than the efficacy of the proposed strategy; therefore, larger studies and randomized experiences are needed.

## 5. Conclusions

A highly mobile endocavitary structure makes PM arrhythmias a unique clinical entity with peculiar features; ideal ablation requirements often relate to precise activation mapping, best pace mapping, stable contact and multiple lesions around the site all together. The combination of intracardiac echocardiography and contact force sensing may overcome the specific difficulties. Furthermore, an integrated approach using ICE and CFS may increase the success rate of PM VA ablation.

## Figures and Tables

**Table 1:** Demographic characteristics and clinical data of the patients.

Variables	ICE/CFS Group (n = 21)	No ICE/CFS Group (n = 12)	Overall (n = 33)	*p* Value
Demographics				
Age (y, Mean ± SD)	48 ± 16	51 ± 13	49 ± 15	0.651
Gender (%male)	71.40%	66.7%	69.7%	1.000
Smoking (%)	42.90%	33.33%	39.40%	0.719
Comorbidities (%)				
Coronary heart disease	42.90%	25.00%	36.40%	0.457
Hypertension	25.80%	16.70%	24.20%	0.678
Duration of symptoms	11.88 ± 14.95	20.55 ± 21.96	15.29 ± 18.16	0.187
Cardiac function				
LVEF	0.57 ± 0.09	0.59 ± 0.07	0.58 ± 0.08	0.823
LVD	52.15 ± 4.90	48.44 ± 5.65	51.00 ± 5.33	0.084
LAD	38.65 ± 5.77	37.78 ± 6.66	38.38 ± 5.96	0.723
Laboratory test				
CNTI (ng/dL)	1.91 ± 1.74	1.60 ± 1.53	1.82 ± 1.66	0.391
CK-MB (ng/dL)	3.13 ± 7.67	0.71 ± 0.21	2.29 ± 6.25	0.938
CK (u/L)	129.57 ± 209.89	60.25 ± 32.13	110.45 ± 180.88	0.069
NT-pro BNP (ng/mL)	478.25 ± 836.15	335.67 ± 278.43	449.73 ± 753.40	0.933
AAD Pre	66.70%	83.30%	72.70%	0.429
AAD Post	76.20%	75.00%	75.80%	1.000

Abbreviations: LVEF, left ventricular ejection fraction; CTNI, cardiac troponin I; CK-MB, creatine kinase-MB; NT-pro BNP, NT-pro B-type natriuretic peptide; LVD, left ventricular diameter; LAD, left atrial diameter; AAD Pre, use of antiarrhythmic drugs prior to ablation; AAD Post, use of antiarrhythmic drugs after ablation.

**Table 2:** Procedural characteristics.

Variables	ICE/CFS Group (n = 21)	No ICE/CFS Group (n = 12)	*p* Value
LV PAP VA target			1.000
Anterolateral PM	7 (33.33%)	4 (33.33%)	
Posteromedial PM	14 (66.67%)	8 (66.67%)	
Location targeted			NA
Tip	5 (23.80%)	NA	
Body	11 (52.38%)	NA	
Base	5 (23.80%)	NA	
Hyperechogenicity on ICE	6 (28.50%)	NA	NA
Type of LV access			1.000
Transaortic approach	14 (66.67%)	8 (66.67%)	
Combined access	7 (33.33%)	4 (33.33%)	
Average contact force (g)	7.52 ± 3.311	NA	NA
Total RF duration (s)	258.24 ± 141.39	302.83 ± 135.80	0.405
Total procedure time (min)	157.38 ± 49.38	158.08 ± 67.88	0.973
Total X-ray time (S)	165.67 ± 47.80	365.00 ± 183.73	<0.001
Lesions	4.90 ± 2.23	10.17 ± 5.89	0.006
Early site (ms)	35.10 ± 5.08	34.25 ± 4.35	0.618
Acute success	100%	66.67%	0.012
Long-time recurrence	14.20%	50.00%	0.044
Cost performance	7864.86 ± 769.79	4519.03 ± 1504.44	<0.001

Values are n (%) or mean ± SD. Abbreviations: Recurrence, reappearance of the same clinical arrhythmia during ablation; RF duration: radiofrequency duration ablation.

**Table 3:** Characteristics of the PVC/VT.

N=	Age	Sex	Clinical	ICE/CFS	Origin	Location	PVC Burden	
							Preprocedure (%)	Postprocedure (%)
Patient 1	34	F	PVC	0	PPM	NA	15.65	11.94
Patient 2	62	M	PVC	0	PPM	NA	20.82	0.95
Patient 3	26	F	PVC	0	PPM	NA	17.98	0.48
Patient 4	56	M	PVC and VT	0	PPM	NA	24.41	0.61
Patient 5	55	F	PVC	0	APM	NA	22.46	13.48
Patient 6	61	M	PVC	0	PPM	NA	16.99	15.12
Patient 7	36	M	PVC	0	PPM	NA	19.43	16.98
Patient 8	52	M	PVC	0	PPM	NA	11.37	2.46
Patient 9	48	M	PVC	0	APM	NA	24.53	11.35
Patient 10	53	M	PVC	0	APM	NA	17.53	0.20
Patient 11	69	F	PVC	0	APM	NA	11.63	1.43
Patient 12	74	F	PVC and VT	0	PPM	NA	13.20	10.37
Patient 13	46	M	PVC	1	PPM	Base	31.20	0.59
Patient 14	63	M	PVC	1	APM	Body	19.95	0.88
Patient 15	59	M	PVC and VT	1	PPM	Body	15.16	1.27
Patient 16	39	M	PVC	1	APM	Tip	20.18	0.66
Patient 17	5F	F	PVC	1	APM	Base	16.00	16.20
Patient 18	42	F	PVC and VT	1	PPM	Body	14.37	0.46
Patient 19	58	M	PVC	1	PPM	Base	17.08	0.15
Patient 20	68	M	PVC	1	PPM	Base	15.09	0.27
Patient 21	14	M	PVC	1	PPM	Body	10.44	0.52
Patient 22	46	M	PVC and VT	1	PPM	Body	37.59	1.17
Patient 23	56	M	PVC	1	PPM	Body	20.92	18.28
Patient 24	45	F	PVC and VT	1	PPM	Tip	20.67	1.16
Patient 25	53	F	PVC	1	APM	Tip	16.82	0.83
Patient 26	69	M	PVC	1	PPM	Body	16.98	1.61
Patient 27	66	M	PVC	1	PPM	Tip	15.24	1.10
Patient 28	60	M	PVC and VT	1	PPM	Body	10.80	0.83
Patient 29	71	M	PVC	1	APM	Body	11.37	1.51
Patient 30	33	F	PVC	1	APM	Tip	12.37	10.32
Patient 31	19	M	PVC	1	PPM	Body	13.10	2.43
Patient 32	22	M	PVC	1	PPM	Base	14.29	1.67
Patient 33	47	F	PVC and VT	1	APM	Body	12.47	1.86

## Data Availability

The datasets generated and analyzed during the current study are not publicly available due data privacy protection and ownership, but are available from the corresponding author on reasonable request.

## Supplementary Materials

The following supporting information can be downloaded at: https://www.mdpi.com/article/10.3390/jcm12093154/s1, Video S1: opposite significance of ICE and CFS indicated; Videos S2–S4: alternative contact directions through different ways.
